# TIMESS a power analysis tool to estimate the number of locations and repeated measurements for seasonally and clustered mosquito surveys

**DOI:** 10.1007/s10479-023-05491-3

**Published:** 2023-07-10

**Authors:** Luigi Sedda, Benjamin M. Taylor, Russell Cain, Élodie A. Vajda, Allison Tatarsky, Neil F. Lobo

**Affiliations:** 1 Lancaster Ecology and Epidemiology Group, Health Innovation One, Lancaster University, Sir John Fisher Drive, Lancaster, LA1 4AT UK; 2 Malaria Elimination Initiative, Institute for Global Health Sciences, University of California San Francisco, 550 16th Street, San Francisco, CA 94158 USA; 3 Eck Institute for Global Health, University of Notre Dame, 4147 Jenkins Nanovic Halls, Notre Dame, IN 46556 USA; 4 School of Mathematical Sciences, University College Cork, Cork, T12 XF62 Ireland

**Keywords:** Mosquito sampling, Temporal autocorrelation, Intra- and between-cluster correlation, Correlated measurements, Surveys

## Abstract

Every day, hundreds of mosquito surveys are carried out around the world to inform policy and management decisions on how best to reduce or prevent the burden of mosquito-borne disease or mosquito nuisance. These surveys are usually time consuming and expensive. Mosquito surveillance is the essential component of vector management and control. However, surveillance is often carried out with a limited if not without a quantitative assessment of the sampling effort which can results in underpowered or overpowered studies, or certainly in overpowered studies when power analyses are carried out assuming independence in the measurements obtained from longitudinal and geographically proximal mosquito surveys. Many free, open-source and user-friendly tools to calculate statistical power are available, such as G*Power, glimmpse, powerandsamplesize.com website or R-cran packages (pwr and WebPower to name few of them). However, these tools may not be sufficient for powering mosquito surveys due to the additional properties of seasonal and spatially clustered repeated measurements required to reflect mosquito population dynamics. To facilitate power analysis for mosquito surveillance, we have developed TIMESS, a deployable browser-based Shiny app that estimates the number of repeated measurements and locations of mosquito surveys for a given effect size, power, significance level, seasonality and level of expected between-location clustering. In this article we describe TIMESS, its usage, strengths and limitations.

## Introduction

Measuring and monitoring mosquito indicators (e.g. abundance, density, composition, biting rate, resistance), and mosquito population dynamics with acceptable accuracy and precision remains a priority in both epidemiological and entomological surveillance and control programs (Zhou et al., 2004). To adequately address research and program questions, mosquito indicators need to be accurately measured over time and space (Reisen & Lothrop, 1999). However, the large majority of mosquito surveys’ sampling effort (the total number of surveyed sample units in space and time during a surveillance campaign) is primarily dictated by accessibility and financial constraints rather than mosquito distribution properties (e.g. spatial and temporal distribution, amount of clustering in space and time). Most published studies on malaria vector ecology use arbitrary sample size and often overlook the statistical consideration (see Liu et al., 2012; Zittra et al., 2017 as examples). In general, mosquito surveillance managers try to address the issues related to seasonality and spatial clustering by applying a subjective approach that recognises the presence of these properties, but without a formal assessment of them. For example, such a subjective approach might entail repeating the surveys every 1, or 2 weeks, or monthly, and considering multiple sampling units in each area (see Sedda et al., 2019 for a short literature overview), even when some information about the local mosquito seasonality and their clustering is available. Therefore, concentrating the sampling effort around or post a rainy season and malaria outbreak may not be sufficient to represent the epidemiological and entomological processes.

Calculating trapping frequency and the number of locations to reflect the temporal seasonality and spatial clustering of the process under study (mosquito infection, insecticide resistance or simply the mosquito distribution), and therefore precisely estimate the effect size for a certain power level is a challenging task due to the lack of dedicated computer programmes. Power is the probability that you will not fail to reject a false null hypothesis, or in simpler words, power is the probability of the study design to estimate the effect size at a pre-defined statistical significance level (Arnholt, 2019). In this work effect size is defined by the ratio between the tested difference, and its standard deviation. Therefore, reliable sampling program for estimating the mosquito population density and their changes should include information about sampling unit, sampling time (time, date and frequency of sampling) and the number of sampling locations. These values must be obtained from spatially- and temporally-corrected power analyses in order to increase the accuracy in the determination of the spatial distribution of the mosquito process and its changes over space and time due to the nature of the processes, current or new interventions, or niche transformation.

Conducting repeated surveys at the same locations, what we will refer as ‘repeated measurements’, implies dependence between measurements, or in other words a correlation between measurements, which if not accounted for in sample size calculations, can result in loss of precision even in presence of oversampling (Bakdash & Marusich, 2017), as for example when oversampling many locations with few repeated measurements. For this reason, repeated measurements can simultaneously increase statistical power for detecting changes while reducing the costs of conducting a surveillance study (a review is provided by Guo et al., 2013). By correcting for the presence of temporal correlation and within-location clustering in the repeated measurements, the sampling effort should successfully maximise the level of confidence and minimise the uncertainty for the reliable identification of the mosquito indicators*.* In a spatial sampling design this can take the form of rolling cross-sectional designs to survey and resurvey large numbers of separate study locations in a logistically feasible manner (Killeen et al., 2021).

With the aim to simplify the use, and support the validity of power analyses of the many mosquito surveillance campaigns conducted every day in the world, we present an algorithm, TIMESS, for performing power analyses in presence of seasonality and within-location clustering in the data. TIMESS is an user-friendly application (for the reduced number of parameters and presence of visualisation tools) implemented as a combined R package (R Core Team, 2022) and Shiny app (Beeley, 2016). The obtained power results can inform sampling effort planning by providing the number of repeated measurements and locations needed to obtain precise and significant effect size estimates. This information is critical for monitoring vector population dynamics and for assessing the efficacy of vector control measures, especially for those settings strained by insufficient resources (since the presence of temporal and spatial correlation can reduce the sampling effort).

## Model

### Null hypothesis

We start describing TIMESS by defining the null hypothesis, which is:

1 $$ {\text{H}}_{0} :x = 0 $$

where *x* is the smallest effect size to be detected. As described above, effect size is the ratio between the tested difference, *x*, and its standard deviation. *x* is expressed in proportion, as for example, the % decrement in a mosquito species after intervention. In case of comparison between two mosquito species/populations, the estimated difference is divided by the mean standard deviation of the effect for each mosquito species/population. This allows the effect size to be comparable in both between-groups designs and repeated-measures designs (Brysbaert, 2019).

### Sample size calculation for aggregated data: Taylor’s power law

Historically (see for example Southwood & Henderson, 2000), a simple approach for determining the sample size, *n*, for an effect size, *x*, detected within a pre-defined precision level, *α*, was based on a t-test:

2 $$ n = \left( {\frac{t}{{Z_{\alpha } x}}} \right)^{2} $$

where *t* is the critical value for the *t* distribution at the fixed type I error, and *Z*_*α*_ is the half-width confidence interval percentile of the standard normal distribution. This approach fails in accounting for explicit aggregation in space and or in time. A more advanced method for natural populations, such as those for mosquitoes (Lindblade et al., 2000), is the Taylor’s power law (Taylor, 1961):

3 $$ n = a\overline{X}^{\;{\left( {b - 2} \right)}} \left( {\frac{t}{{Z_{\alpha } x}}} \right)^{2} $$

which requires the knowledge of three parameters: sample mean, $\overline{X}$, a parameter describing the degree of aggregation of the species in the environment, *b*, and a scaling factor, *a* (Routledge & Swartz, 1991). The last two parameters are usually estimated by linear regression analysis of log-transformed sample means against log-transformed variance of abundance (Zhou et al., 2004). When spatial distribution of units (e.g. mosquitoes) is random, variance and mean are equal and the aggregation parameter is equal to 1 (equivalent to a Poisson distribution). There are various criticisms on Taylor’s power law, on both the interpretation of its parameters and their validity (Routledge & Swartz, 1991), and specifically for our study, in the limitation of Taylor’s power law to account for explicit seasonal-dependent measurements due to mosquito aggregation changes over time (Zittra et al., 2017).

### Sample size calculation for seasonal data

Mosquito surveillance relies on repeated measures which increase the power to detect the effect size (Bakdash & Marusich, 2017), and at parity of repeated measurements, the power increases with increasing correlations between repeated measurements. Following the notation of Lui and Cumberland (1992) for temporally correlated data, the sample size for a desired power 1 − *β* (with *β* the type II error rate) at a given *α* precision level can be obtained from:

4 $$ n = \frac{2}{{x^{2} }}\left[ {\sigma_{b}^{2} + \frac{{\sigma^{2} }}{k}\left( {1 + \left( {k - 1} \right)\rho_{\Delta } } \right)} \right]\left( {Z_{\alpha /2} + Z_{\beta } } \right)^{2} $$

where $\sigma_{b}^{2}$ and *σ*^2^ are the variances for the effect among sample units and errors of repeated measurements respectively, *k* is the number of repeated measurements, *ρ*_*Δ*_ is the correlation among repeated measurements (see below), and *Z*_*α*/2_ and *Z*_*β*_ are the upper *α*/2 and *β* percentiles of the standard normal distribution respectively. Variance of *x,* correlation parameter, power and precision level must be provided, and TIMESS uses the adjustment proposed by Zhang et al. (2018) for the calculation of the sample size when *σ*^2^ is unknown. This adjustment is based on the formula to estimate effect size in one-way design with the test statistic following an exact F distribution.

### Sample size calculation for spatially aggregated and seasonal data: TIMESS

**Fig. 1:**
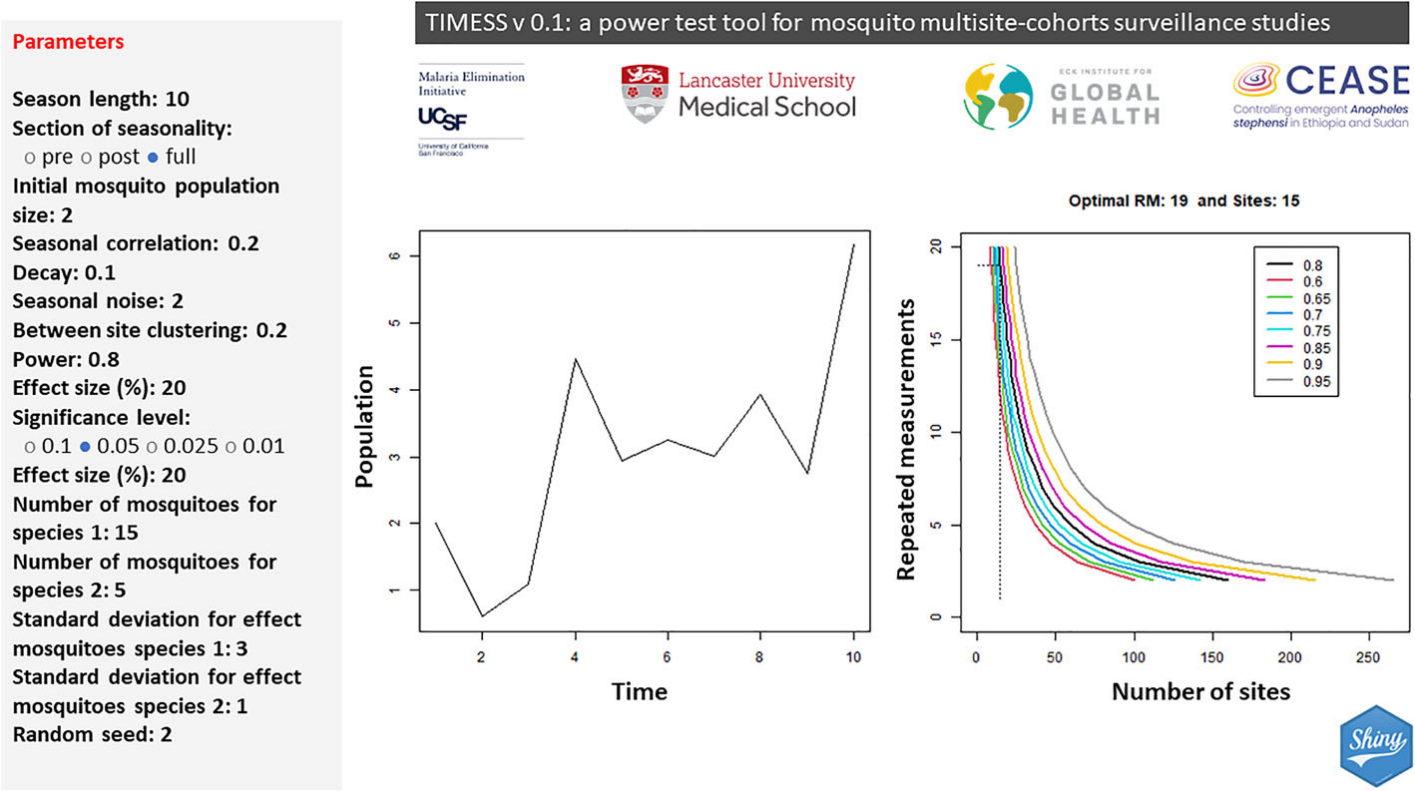
Simplified screenshot of TIMESS R-shiny application. Used parameters: *k* = 10, *N* = 2, *θ* = 0.1, *ρ* = 0.2, seasonality noise = 2, between site clustering *ρ*_*c*_ = 0.2, power = 0.8, *x* = 20, *α* = 0.05, number of mosquito species = 1, number of mosquitoes for species 1 = 15, standard deviation of the number of mosquitoes for species 1 = 3, random seed = 2. The population/time graph shows the selected seasonality, while the number of sites/repeated measurements graph shows the power curves at various power levels

In TIMESS, the correlation *ρ*_*Δ*_ is obtained from a dampened autoregressive function, which in comparison to an autoregressive function, allows for a smoother decline of the correlation between measures farther apart in time (Morgan & Case, 2013). Within a dampened autoregressive model, the correlation for a given time difference *Δ* is:

5 $$ \rho_{\Delta } = \rho^{{\left( {k - 1} \right)^{\theta } }} $$

where *ρ* is the correlation of the values (in TIMESS named as ‘seasonal correlation’), and *θ* the dampening factor. In TIMESS users can visualise the changes in seasonality by tuning these two parameters thanks to a visualisation tool (Fig. 1 top left graph).

TIMESS searches for the optimal sampling effort that satisfies the pre-determined seasonality and location clustering for a grid of repeated measurements and number of locations, by minimizing the difference between the sample size in Eq. (4) [which seasonality is expressed by Eq. (5)] and the sample size in Eq. (6) [which clustering is expressed by Eq. (7)].

Clustering in the measurements between locations is defined as the amount of measurement variation that can be explained between locations. TIMESS accounts for measurements clustering within and between location as in a cluster randomised trial (Raudenbush, 1997), where each cluster is a location. With between cluster correlation *ρ*_*c*_, the number of locations, *n*_*c*_, necessary to precisely detect the effect size at a desired power 1 − *β* and significance level *α*, with *m* units (mosquitoes) in each cluster or location (therefore full sample size is obtained by the product of *n*_*c*_ and *m*) is:

6 $$ n_{c} = \frac{2}{{x^{2} }}\sigma_{T}^{2} \left[ {\left( {1 + \left( {m - 1} \right)\rho_{c} } \right)} \right]\left( {Z_{\alpha /2} + Z_{\beta } } \right)^{2} $$

with

7 $$ \rho_{c} = \frac{{\tau^{2} }}{{\sigma_{T}^{2} }} $$

where the quantity (1 + (*m* − 1) *ρ*_*c*_) is also known as the variance inflation factor (VIF) (Hemming et al., 2011), $\sigma_{T}^{2}$ is the total variance in the outcome, * τ*^2^ is the between clusters (locations) variance. The total variance is given by the sum of the between clusters and within clusters variance. The intra-cluster correlation, *ρ*_*c*_, represents the proportion of variance due to between location variation. If *ρ*_*c*_ is 0 the variance is fully explained by variation within location and there is independence between the units within the location. If *ρ*_*c*_ is 1 all the variation is due to differences between locations and the units within each location are identical (Rutterford et al., 2015). Apart from Eq. (6), *ρ*_*c*_ can be calculated using other methods (see Zhang et al., 2018). The use of a parameter representing the correlation between observed individuals in each cluster (sub-villages, villages or other geographic or population subunits) is a common practice in mosquito sampling design studies (Hayes & Bennett, 1999; Killeen et al., 2021).

Finally, the optimal number of locations and repeated measurements is given by the value of *k* that allows the approximate equivalence in:

8 $$ n_{c} m \approx nk $$

In TIMESS *ρ*_*Δ*_ is obtained from Eq. (5) above, and *ρ*_*c*_ is user defined with low values associated to high clustering because most of the variance is explained within location.

TIMESS is made available in a Shiny web-app (Beeley, 2016) (see software availability section).

## Results

The shiny-web app interface for TIMESS is shown in Fig. 1. The parameters required by TIMESS are (in brackets the simplified names of the parameters as shown in the shiny app interface):

I. mosquito seasonality pattern parameters: maximum *k* value (repeated measurements or season lengths in time units), initial mosquito population size, *N*, to help in visualising a realistic seasonal curve (Fig. 1 population/time graph), seasonal correlation *ρ* (rho), and the decay *θ* (theta, dampening factor);
II. power analysis parameters: the power (1 − *β*), *α* significance level (four levels available 0.1, 0.05, 0.025, 0.001), and minimum difference to be detected expressed in percentile, effect size *x*, and its standard deviation (or standard deviations when comparing two mosquitoes) ‘standard deviation for effect mosquito species 1’ and ‘standard deviation for effect mosquito species 2’;
III. mosquito-relevant parameters: number of species (one or two) for two-sample comparison, and *m* (number of mosquitoes per site per measurement per species, simply ‘number of mosquitoes for species 1’ and ‘number of mosquitoes for species 2’);
IV. spatial clustering parameter: between site clustering, *ρ*_*c*_;
V. randomness and noise parameters: seasonal noise (variance in the values of the mosquito population due to the stochastic nature of mosquito populations) and random seed used for ensuring repetition of the same seasonality curve due to added random seasonality noise; therefore the random seed does not affect the power results but only the visualisation of the seasonal curve.

**Fig. 2:**
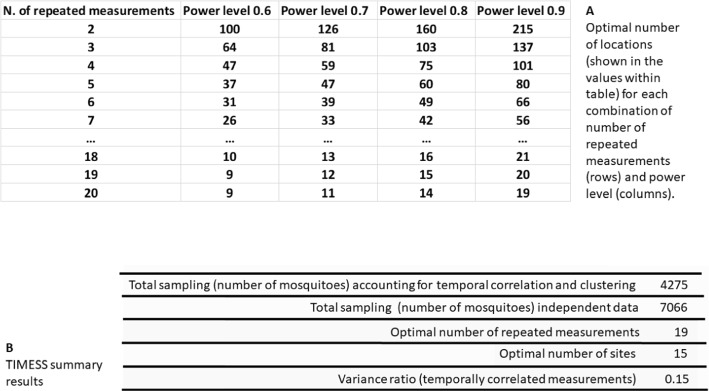
**A** table of optimal number of sites at different power levels and number of repeated measurements that can be download from TIMESS (values for repeated measures from 8 to 17 are hidden for figure readability). **B** Summary results from the power analysis. Used parameters: *k* = 10, *N* = 2, *θ* = 0.1, *ρ* = 0.2, seasonality noise = 2, between site clustering *ρ*_*c*_ = 0.2, power = 0.8, *x* = 20, *α* = 0.05, number of mosquito species = 1, number of mosquitoes for species 1 = 15, standard deviation of the number of mosquitoes for species 1 = 3, random seed = 2

Apart from the seasonal and power curves graphs shown in Fig. 1, results from TIMESS can be download in form of table (csv format), where for each power level and number of repeated measurements, the optimal number of locations is provided (Fig. 2 panel A).

Summary results (Fig. 2 panel B) report the optimal number of repeated measurements and locations, the total sampling in terms of number of mosquitoes that need to be collected when considering temporal correlation and clustering, and when collections are considered independent (total sampling for independent data). As expected, the total sampling is lower in presence of correlated and highly clustered data, it requires almost 61% the number of mosquitoes required in case of assuming independent measurements. The latter is calculated from the power analysis based on a two tailed t-test for two samples [Eq. (2)]. Finally, the variance ratio (VR) indicates the amount of variance of the test with correlated repeated measurements, i.e. 1 − VR is the reduction in variance in comparison with a test with two independent means (Morgan & Case, 2013).

**Fig. 3:**
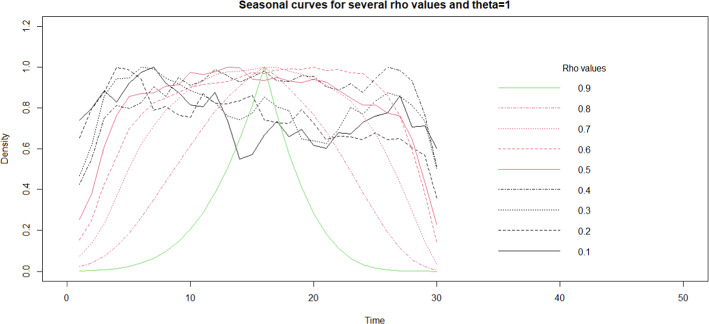
Effect of rho parameter on the seasonality curve within a period of 30 time units and a seasonality noise of 2 while theta fixed to 1. The starting mosquito population was 100

**Fig. 4:**
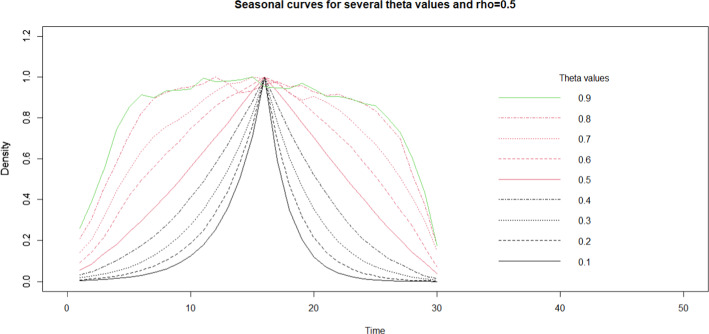
Effect of theta parameter on the seasonality curve within a period of 30 time units and a seasonality noise of 2 while rho fixed to 0.5. The starting mosquito population was 100

Most of TIMESS parameters are conventional of any power analysis (see for example http://powerandsamplesize.com). In TIMESS the user has the advantage to visually draw and include the mosquito seasonality by inputting the values for the parameters rho, *ρ*, and theta, *θ*. In Figs. 3 and 4 the standardised seasonality curves for rho, when theta is fixed at a value of 1 (Fig. 3) and theta, when rho is fixed to a value of 0.5 (Fig. 4) are shown.

The effect of these curves (and relative parameters) on the optimal number of repeated measurements and locations is shown in Table 1.

**Table 1:** Optimal numbers of repeated measurements and locations by correlation factor rho and dampening factor theta

Rho (*ρ*)	Theta (*θ*)	Optimal number of repeated measurements	Optimal number of locations	Sampling effort
0.1	1	19	15	285
0.2	1	18	16	288
0.3	1	18	16	288
0.4	1	17	17	289
0.5	1	16	18	288
0.6	1	15	19	285
0.7	1	14	21	294
0.8	1	13	22	286
0.9	1	12	24	288
0.5	0.50	15	19	285
0.5	0.75	15	19	285
0.5	1.00	16	18	288
0.5	1.25	17	17	289
0.5	1.50	18	16	288
0.5	1.75	18	16	288
0.5	2.00	18	16	288
0.5	2.25	18	16	288
0.5	2.50	19	15	285

All the rest of the parameters are fixed as: between site clustering *ρ*_*c*_ = 0.2, power = 0.8, *x* = 20, *α* = 0.05, number of mosquito species = 1, number of mosquitoes for species 1 = 15, standard deviation of the number of mosquitoes for species 1 = 3, random seed = 2. Sampling effort is defined as the product of repeated measurements and number of locations

Table 1 shows that the change in seasonality at parity of clustering—i.e. the ratio between variance explained within and between locations, does not affect the sampling effort, which stays on similar values, but only the number of repeated measurements and number of locations. Generally, as theta increases the required repeated measurements increases due to measurements becoming independent (Fig. 4 and Table 1 bottom half). Conversely, larger rho increases dependence between measurements and therefore reduces the number of repeated measurements needed (Fig. 3 and Table 1 upper half).

## A primer on how to use TIMESS and its outputs

### First step: produce a seasonal curve

TIMESS users need first to decide the seasonality shape and size of the mosquito species (or the common seasonality shape and size for two mosquito species analysis). To do so, the user needs to modulate four parameters: seasonal length, initial mosquito population size, seasonal correlation, and the decay. The most challenging choice is most likely to be fixing the values for the seasonal correlation and decay. This choice requires careful consideration, in fact small variation of these parameters could have a significant effect on the number of mosquitoes to be collected. To guide the users, here we distinguish between two scenarios: (i) previous data is available, and therefore the parameters can be estimated quantitatively; or (ii) previous data is missing, thus the parameters’ values are decided through a trial-and-error approach or adopting conservative assumptions.

If previous mosquito data is available (for same species and location, ideally from the last 4–5 years), the user can input directly the autocorrelation function (ACF) value (Wylomanska, 2012). To use this value, the user must set theta to 0 and input the ACF value in rho. ACF calculation is available in most statistical software/packages (for example in R within the nmle package, Pinheiro et al., 2017). With this approach *ρ*_*Δ*_ = ACF [see Eq. (5)].

In the absence of available data, the user can try to replicate the seasonality curve from the literature and by trial-and-error approach. In this case, Figs. 3 and 4 can help with deciding the initial values for these parameters. If no data exists, the seasonal curve choice can rely on expert opinion with again a trial-and-error approach on turning the parameters to obtain a plausible seasonal curve.

If none of these options above are available, assuming a conservative rho = 0, i.e. no correlation between values, need to be preferred than assuming any correlation at all—the latter will always reduce the sample size with the risk of underpowering the study. In other words, rho = 0 is equivalent to the assumption of fully independent data. Note that when rho = 0 any values in theta will not be effective. The interval for rho is 0 and 1: $\rho \in [{0,1})$; and for theta any positive number: *θ* $\in [0,+\infty )$.

The initial population size parameter, *N*, is only used for visualisation in order to reproduce a seasonal curve with similar or identical size from literature. Therefore, the parameter *N* does not affect the number of mosquitoes needed or the optimal number of repeated measurements and locations.

Finally, the unit of the time series (which total length is *k*) must be the same as the measurement unit to be repeated. For example, if the surveillance is planned to be monthly, then *k* must be in months. The length itself should encompass the full seasonality of the species or the full length of the surveillance, whichever is the shorter. By default, TIMESS produces optimal repeated measurements for 2*k units, in order to consider sub-unit frequency. For example, if *k* = 10 months and the optimal number of repeated measurements is 15, then the frequency for the measurements should be every 20 days (to reach 15 measurements within 10 months).

### Second step: select the amount of spatial clustering

The parameter ‘between site clustering’ controls for the ratio of variance explained within site by the seasonality and between site by spatial autocorrelation. A large value means that most of the variance is explained across locations instead of within location. Therefore at parity of seasonal correlation, increasing this value increases the number of locations needed and the number of mosquitoes needed, which can exceed the number of mosquitoes needed by simply performing a t-test for two samples (historical approach). For example, a between site clustering of 0.5 will require three times more mosquitoes than the one assuming a simple comparison of means with independent data.

As in the first step, setting the between site clustering requires a knowledge or assumptions around the amount of spatial autocorrelation or spatial similarity between locations in regarding to the effect size. In presence of data, areal or point, global or local pattern analyses will allow for the calculation of the spatial clustering. Aldstadt (2010) proposes various measures to calculate the amount of clustering in the data (Getis & Ord, 1992).

In the absence of previous data, a qualitative assessment of the potential spatial clustering should be carried out; this spatial clustering should consider the ecological homogeneity of the area and the expected average distance between locations (especially if a coverage surveillance is planned) (Dubes & Zeng, 1987). Therefore, large distances and great ecological heterogeneity will imply a low level of global clustering, and vice versa, low distances and homogeneity will imply a high level of global spatial clustering (Fortin et al., 2002).

If the user is unsure on the level of spatial clustering, then, as for step one, a conservative strategy should be adapted by selecting a high level of between site clustering—with the warning that the number of mosquitoes required can increase substantially in heterogeneous mosquito populations.

The interval for between site clustering, *ρ*_*c*_, is: *ρ*_*c*_ $\in \left[{0,1}\right].$

### Third step: decide the effect size

A power analysis needs to have a well formulated statistical hypotheses (the null and alternative). We refer to the large amount of literature for how to best formulate hypotheses in the context of power analyses (Banerjee et al., 2009). The choice of effect size must be carried out carefully since its value will depend on the significance level and the standard deviation(s) (Brysbaert, 2019; Sawilowsky, 2009).

In TIMESS the effect size is the expected change during an experiment which is often a comparison (number of mosquitoes before and after an intervention, or number of mosquitoes outside and inside a household etc.). It is expressed in the % in variation of number of mosquitoes, e.g. a reduction of y% or a difference of z%. It is also important to identify the variation around the effect size (parameter: standard deviation for effect mosquito 1 in a case of a single species). The effect size is based on the alternative hypothesis, and it is associated to an entomological significant effect instead of a statistically significant effect. The choice of effect size can be guided by similar studies (e.g. a published amount of mosquito reduction after an intervention), but in this case, careful consideration of the sample size used in the original research is required, as well as if it was the result of multiple tests (which inflates the significance level). It is likely that many of the published effect sizes are large because large effect sizes are more likely to be significant (Brysbaert, 2019).

At the time of the present article, only a handful of studies for mosquito surveillance report the effect size used for powering their study (e.g. Wamaket et al., 2021), therefore it is likely that limited support will come from literature. If attributing a value to the effect size is resulting challenging, the user is prompted to consider a value within the categories proposed by Cohen (1988), who distinguished between three types of effect sizes: 20% for a small effect size, 50% for a medium effect size, and 80% for a large effect size. However, we warn the users that while Cohen’s categories are commonly used in ecological studies, they originated from psychological studies, hence validity of the use of these categories needs to be assessed and justified.

### Fourth step: answer the question on how many mosquitoes can be collected by each trap

The parameters ‘Number of mosquitoes for species 1’ and ‘Number of mosquitoes for species 2’ are the expected number of mosquitoes collected on average by a single trap and a single measurement event. These parameters do not influence the effect size, but they determine the number of repeated measurements and locations.

These values should be based on the type of trapping method (Murindahabi et al., 2022) and on the local mosquito population size. They are usually obtained from previous entomological field work in the same area or from the literature. Although mosquito count data are not available at the country level across Africa, with the exception of bionomics (Massey et al., 2016) and species occurrence (Kyalo et al., 2017), many local and regional studies can instead be used. One of these studies is a recently published historical dataset (from 1947 to 2016) on various *Anopheles* spp. collected in East and Southern Africa coast (Bartilol et al., 2020). They reported an average of 22 *Anopheles arabiensis* (standard deviation 80) and 13 *Anopheles gambiae s.s.* (standard deviation 33) caught monthly.

In absence of any previous information, a pilot study on few locations is recommended. For example, by starting the surveillance in few locations (two or three) in the area of interest, for 1 or 2 months, and then use the field data obtained as input in TIMESS.

### Fifth step: select power and significance level

A brief introduction on power and significance level is necessary to understand the importance of these two parameters in sampling size calculations. First, the significance level is the probability of type I error (rejecting the null hypothesis when the null hypothesis is true) that the user is taking as risk. Type I error is often referred as *α*, and 1 − *α* is the precision. Power represents the probability of not making a type II error (not rejecting the null hypothesis when the null hypothesis is false); in other words, the probability of observing an effect equal or larger than the pre-defined effect size. Power is obtained by 1 − *β*, where *β* is the type II error.

Many studies use the conventional 0.05 for *α* and 0.2 for *β* which is the well-known five-eighty convention proposed by Cohen (1988) (where eighty is the 80% when *β* is expressed in % and power is 100 − *β*). This is reinforced by other authors for any biological and medical studies (Bausell & Li, 2002). However, the Cohen five-eighty convention was set for psychological studies, in which risks and assumptions are very different from those associated to ecological studies, meaning relative costs of type I and type II errors should be considered (di Stephano, 2003).

Type I error, or significance level, should not be influenced by the fact that most of the studies use a 0.05 significance level (Columb & Atkinson, 2016). Increasing this level will increase the likelihood of rejecting the null hypothesis, thereby concluding that there was an effect equal or larger than the pre-defined effect size. However, this result could be simply due to the inherent variation in the data. Conversely, a very small significance level will favour large effect size only (Morrison et al., 2008).

Significance level and power are intrinsically dependent, whereby decreasing type I error will inevitably increase the risk of type II error (Cohen, 1982). Power is increased by choosing a less stringent significance level or by increasing the effect size (Bausell & Li, 2002). Balancing alpha and beta is therefore the priority of the user (Banerjee et al., 2009). The risk of making the two errors must be based on the risks associated with the research being conducted: financial, health etc. (Sanderson & Petersen, 2002). Recent studies highlight pitfalls associated when considering these two parameters individually, noting that the ratio between them should be taken into consideration (see next step). Alternatively, a conservative approach should be taken by selecting a smaller than anticipated effect size and larger than expected variability while fixing the significance level to 0.1 or 0.05 (Bagiella & Chang, 2019).

### Sixth step: making the most of TIMESS outputs

To help the user to make an informed decision on the sampling effort, TIMESS will compare eight power levels in addition to the one defined by the user: 60%, 65%, 70%, 75%, 80%, 85%, 90% and 95%. Therefore, for the same significance level, the user can decide on the suitable power in post-processing aided by the table shown in Fig. 2A. Here the optimal number of locations and repeated measurements must be evaluated considering financial (including accessibility) constraints and health priorities (e.g. a malaria hotspot or an outbreak). The cost of a sampling design can be expressed as the total time effort needed and the costs associated with this design to it to detect an effect of a given magnitude.

A way to include the risks in the choice of the sampling effort through power and significance level parameters is to consider the ratio between them (Field et al., 2005), which reflects the relative costs of the type I and II errors. For example, the conventional five-eighty will imply that the user is accepting the risk to reject a true effect (type II error) four times more (0.2/0.05) than accepting a false effect (type I error) (Perugini et al., 2018). Field et al. (2007) simulation studies for monitoring purposes found that a ratio of five or greater than five is unlikely to be optimal and smaller ratios should be adopted. Ideally, the costs of type I and type II errors should be known to inform the sampling effort. In the absence of such information (Field et al., 2007) suggest to set the two errors equal to one another.

### Final notes

Mosquito catches are notoriously noisy (stochastic). Tuning the seasonality noise will allow for closer to real seasonal curves which is useful when trying to replicate seasonality curves from the literature. It is advised to set this parameter to 0 when no real data is available, and to appreciate the signal of the seasonality.

Finally, upon TIMESS launch, it will generate seasonal and power curves using default values. However, these default values should never be used as they are not derived from field observations or expert opinions. Therefore, users are cautioned to modify these parameters based on the above considerations and the availability of existing field data.

## Discussion

There is still a lack of guidance for the spatial sampling design of vector sampling in official protocols. For example the ECDC Vector Sampling Field Protocol (https://www.ecdc.europa.eu/sites/default/files/documents/Vector-sampling-field-protocol-2018.pdf) does not provide information on how many repetitions should be carried out in presence of seasonal vector dynamics and neither on the number of locations required with spatially clustered data.

In general, mosquito surveillance requires a multitude of decisions which include trap types, collection approaches (e.g. indoor or outdoor) and sampling design (Wilke et al., 2022). While these decisions are supported by experimental evidence, the latter is complicated by the limited number of studies and operative guidance.

Mosquito surveillance data are used to guide major and very expensive vector control decisions in-country, therefore under-powered surveillance designs can limit the robustness of the data and subsequent analyses to drive the decision-making process. Power analyses based on the assumption of independence in measurements can inflate the degrees of freedom of the statistical test since mosquito counts are temporally and spatially correlated. At the same time, assuming homogeneity over space can reduce the degrees of freedom and lead to misrepresentation of the study population. Confounding this relationship is the seasonality, a fundamental process in mosquito population dynamics (Whittaker et al., 2022). For these reasons we have designed a power tool that provides optimal sample size for seasonal and clustered measurements based on prior information and assumptions, and that can support correct powering of the study (Morgan & Case, 2013) when comparable data is limited or not available (for which a model-based approach, frequentist or Bayesian, may be preferred).

TIMESS results have shown that the presence of spatial and temporal autocorrelation has profound financial consequences on the sampling design, i.e. inefficiently using the funding in an underpowered study—if the assumption is geographic homogeneity in the process under study when in fact is a strong spatial heterogeneity; or spending too much when overpowering—as for example when collections within the seasonality period are assumed independent (Rhodes & Jonzén, 2011).

There is little information on current mosquito surveillance campaigns—and therefore a comparison with the values returned by TIMESS is not feasible. In our simulations, weekly surveys appear to not be necessary to observe changes in species composition and density for species with a strong seasonality, as long as the study is repeated over space (using different households in the same locations) as well as over-time. The information provided by TIMESS can help mosquito surveillance managers to combine TIMESS results with cost functions (Ferguson et al., 2014; Kermorvant et al., 2021).

TIMESS adjusts the sample size obtained from an within-effect ANOVA test for repeated measurements (an extension of the paired t-test for more than two groups) (O'Brien & Kaiser, 1985), to clustering effects (Raudenbush, 1997). Correlation between repeated measurements is modelled as structured temporal correlation which assumes the existence of particular correlation patterns among the repeated measures (Guo et al., 2013). In case of pre-existing data availability, the assessment of the correlation within and between locations can be provided by existing packages, such as rmcorr in R (Bakdash & Marusich, 2017), although to our knowledge no tools are available within these algorithms to construct seasonality curves and integrate them in a power test.

One main limitation of this work is the absence of an explicit correction for spatial dependence/autocorrelation since we have focused mainly on the repeated measurements component of the surveillance which may guarantee productivity in trapping (Brown et al., 2011). Spatial dependence can be included using a model-based geostatistical method (Sedda et al., 2019) or assimilating the measurements to a Poisson point-process (Serinaldi, 2013). In addition, we have assumed separability in the variance represented by temporal autocorrelation and within-location variance that may not be appropriate when mosquito variability changes over time (temporal non-stationarity variance) (Whittaker et al., 2022). It is clear, however, that TIMESS and the other methods presented here are not able to model seasonality as a process (seasonal curve instead of a single correlation parameter) including its intensity and pattern variation from year to year. This may be approached via power analyses based on mixed models (Sims et al., 2006) instead of tests, although this again requires a sufficient amount of available data. Finally, we have ignored the effect of environmental drivers on mosquito abundance, such as temperature and rainfall, that could be included by employing Poisson regressions, ARMA models or multinomial frameworks (Zhang & Stern, 2009). This is what we are currently working on for the next version of TIMESS.

## Conclusions

A large body of literature is available for the determination of the sample size in repeated measurements studies (a review is provided by Roy et al., 2007). However, current software/algorithms do not provide tools to visualise and parameterise seasonality that feeds into the power analysis. This is a first step towards power analyses methods for mosquito surveillance that accounts for temporal correlated data (seasonality) and clustering within sites. By providing a quantitative answer to the question of how often one should sample and over many locations, this work contributes to making more intentional decisions on sample effort.

## Data Availability

The deployable TIMESS software is available via Lancaster University PURE repository (pure.lancs.ac.uk) with https://doi.org/10.17635/lancaster/researchdata/595 under CC BY licence.
